# Altered N-Linked Glycosylation in Follicular Lymphoma and Chronic Lymphocytic Leukemia: Involvement in Pathogenesis and Potential Therapeutic Targeting

**DOI:** 10.3389/fimmu.2017.00912

**Published:** 2017-08-02

**Authors:** Nurit Hollander, Joseph Haimovich

**Affiliations:** ^1^Department of Clinical Microbiology and Immunology, Tel Aviv University, Tel Aviv, Israel

**Keywords:** B-cell malignancies, B-cell antigen receptor, immunoglobulin, high-mannose glycans, lectins

## Abstract

B-cell antigen receptor (BCR) expression is indispensable for survival of most B-cell malignancies. In follicular lymphoma (FL), N-linked glycosylation sites are introduced in the immunoglobulin (Ig) variable region genes. Oligosaccharides added to the acquired sites are unusually of the high-mannose type. These glycans interact with mannose-specific lectins, especially with dendritic cell-specific intercellular adhesion molecule-3-grabbing non-integrin (DC-SIGN). Lectin binding to FL triggers persistent activating signals, suggesting that lectins within the tumor microenvironment promote cell survival and proliferation. Insertion of N-glycosylation sites in Ig variable region genes has been detected in other germinal center-associated lymphomas, specifically in subsets of diffuse large B-cell lymphomas and Burkitt’s lymphomas, suggesting involvement of altered glycans in pathogenesis of these malignancies as well. Furthermore, the BCR in chronic lymphocytic leukemia (CLL) carries high-mannose oligosaccharides, albeit in the heavy chain constant rather than variable region. The high expression level of the unique glycoform, particularly in the more aggressive unmutated CLL subset, suggests a functional significance for this glycan in CLL. As lectin interaction with the BCR is critical for FL and probably for some other lymphomas, targeting this interaction is considered to be an interesting therapeutic strategy. Reagents for blockade of lectin–BCR interaction may include antibodies against high-mannose glycans and mannose-based oligosaccharide mimics or non-carbohydrate glycomimetics. Moreover, as this interaction triggers signaling pathways similar to those demonstrated for BCR engagement by antigen, BCR signal transduction inhibitors may emerge as effective therapeutics for lectin-driven malignancies.

## Introduction

Aberrant glycosylation of surface glycoproteins and glycolipids is a hallmark of cancer (1, 2). Alterations in both N-linked and O-linked glycans have been associated with tumor initiation and progression due to their effects on cell–cell and cell–extracellular matrix interactions, cell growth, apoptosis, and cell death. While some changes, such as altered sialylation, are broadly expressed on various malignancies, others are confined to certain cancer types. This mini review presents an overview of aberrant N-linked glycosylation in B-cell lineage malignancies, in which changes in N-linked glycosylation of surface immunoglobulin (Ig) result in the unusual surface expression of high-mannose oligosaccharides.

Normal N-linked glycosylation is commonly carried out in the endoplasmic reticulum (ER) and in the Golgi apparatus (3). In the ER, a core glycan structure is transferred to an asparagine residue located in the consensus sequences Asn-X-Ser or Asn-X-Thr of nascent polypeptides. Following further processing, the fully folded molecules are transported to the Golgi apparatus as glycoproteins carrying high-mannose glycans that terminate in mannose moieties. During transport through the Golgi complex, the glycans undergo further modifications that are catalyzed by glycosyltransferases and glycosidases, giving rise to mature oligosaccharide structures that reach the cell surface as complex-type or hybrid-type glycans. Although this is the conventional pathway in eukaryotic cells, few exceptional glycoproteins reach the cell surface with glycan structures of the high-mannose type that is normally restricted to the ER (4). The latter are transported to the plasma membrane either in a Golgi-independent manner or alternatively by a Golgi-dependent pathway, whereby the molecules cross the Golgi network too fast or with a three dimensional structure that hinders accessibility of glycosylation sites to the appropriate enzymes. Thus, the high-mannose oligosaccharides cannot be fully processed. As described below, the B-cell antigen receptors (BCRs) in several B-cell malignancies carry high-mannose oligosaccharides. The latter may interact with mannose-binding lectins in the tumor microenvironment and initiate antigen-independent signaling that may drive tumor growth/survival. Hence, they may represent a potential target for therapeutic intervention.

## Follicular Lymphoma (FL)

Follicular lymphoma is a human B-cell malignancy that arises in germinal centers and maintains features of its normal counterpart. Expression of surface Ig and maintenance of its signaling activity even in the absence of antigen are indispensable for normal B-cell survival (5, 6). Likewise, expression of surface Ig appears to be critical for the majority of B-cell malignancies because surface Ig-negative tumors are rare even under the selective force of anti-idiotype antibodies (7). Retention of surface Ig in FL is particularly striking since one Ig allele is already disrupted by the t(14;18) chromosomal translocation, which is a hallmark of this malignancy (8). In addition, despite accumulation of point mutations in the variable Ig genes of FL due to ongoing somatic hypermutation, mutational analysis revealed selection for BCR integrity (9). However, although Ig expression is retained amid ongoing mutational activity, it is not clear whether it can respond to altered antigen specificities in order to support tumor growth and if so, what are the multiple antigens involved. There are two main schools regarding the nature of the BCR stimulants in FL. According to the first, self-antigen recognition provides survival signals to tumor cells. Several such autoantigens have been demonstrated, among which are vimentin and myoferlin (10, 11). The second infers that high-mannose glycans, known to be inserted in Ig variable regions of the majority of FLs, impair BCR specificity and affinity to the cognate antigen (12). However, as discussed below, these glycans bind mannose-specific lectins that provide a persistent antigen-independent activating signal (13–15).

The ongoing somatic mutation in FL results in introduction of the motifs Asn-X-Ser/Thr in the Ig variable region genes, most commonly in the CDRs of the heavy chains and less frequently in the light chains (16–19). These novel sites are uncommon in non-functional V_H_ genes, indicating their positive selection in FL. They are present in essentially all FL at diagnosis (16, 19) and are acquired at early stages of lymphomagenesis, as evident by their detection in FL *in situ* (20). Most importantly, they function as acceptor sites for N-linked glycosylation. Strikingly, however, glycans at these sites terminate in mannose moieties, indicating that they do not fully mature in the Golgi apparatus, most probably due to their inaccessibility to the appropriate Golgi enzymes (17, 18). This is remarkable because oligosaccharides are normally located in Ig constant regions, not in the antigen-binding site of the BCR, and are normally complex-type glycans, as expected for surface glycoproteins. The finding that introduced N-glycosylation sites exist in FL but are not frequently detected in normal B cells (16, 21), suggested their involvement in tumor pathogenesis.

The presence of high-mannose glycans in surface Ig elucidates the mechanism by which surface Ig may activate the malignant cells even in the absence of antigen, hence promote tumor progression. Mannosylated Igs of FL interact with C-type lectins, including dendritic cell-specific intercellular adhesion molecule-3-grabbing non-integrin (DC-SIGN) that is expressed by dendritic cells and macrophages. DC-SIGN binding to FL cells triggers BCR aggregation, intracellular Ca^2+^ increase, sustained phosphorylation of the kinases SYK, AKT, PLCγ2, and ERK1/2, and increased expression of cMYC (13–15). DC-SIGN is overexpressed on dendritic cells and macrophages in FL samples. *In situ*, contact of CD68^+^DC-SIGN^+^ cells with CD20^+^ FL cells has been demonstrated in paracortical lymphatic sinuses and in perifollicular areas (15). Moreover, M2 macrophages, known to strongly express DC-SIGN (22), triggered DC-SIGN-dependent FL cell activation as well as abrogation of FL apoptosis (15). Interestingly, a high level of tumor-associated macrophages has been correlated with a bad outcome in FL patients treated with chemotherapy (23). In addition, DC-SIGN is expressed by lymphatic endothelial cells, suggesting that it may contribute to FL dissemination (15). All these indicate how FL cells may exploit their microenvironmental niche for tumor progression.

**Figure 1:**
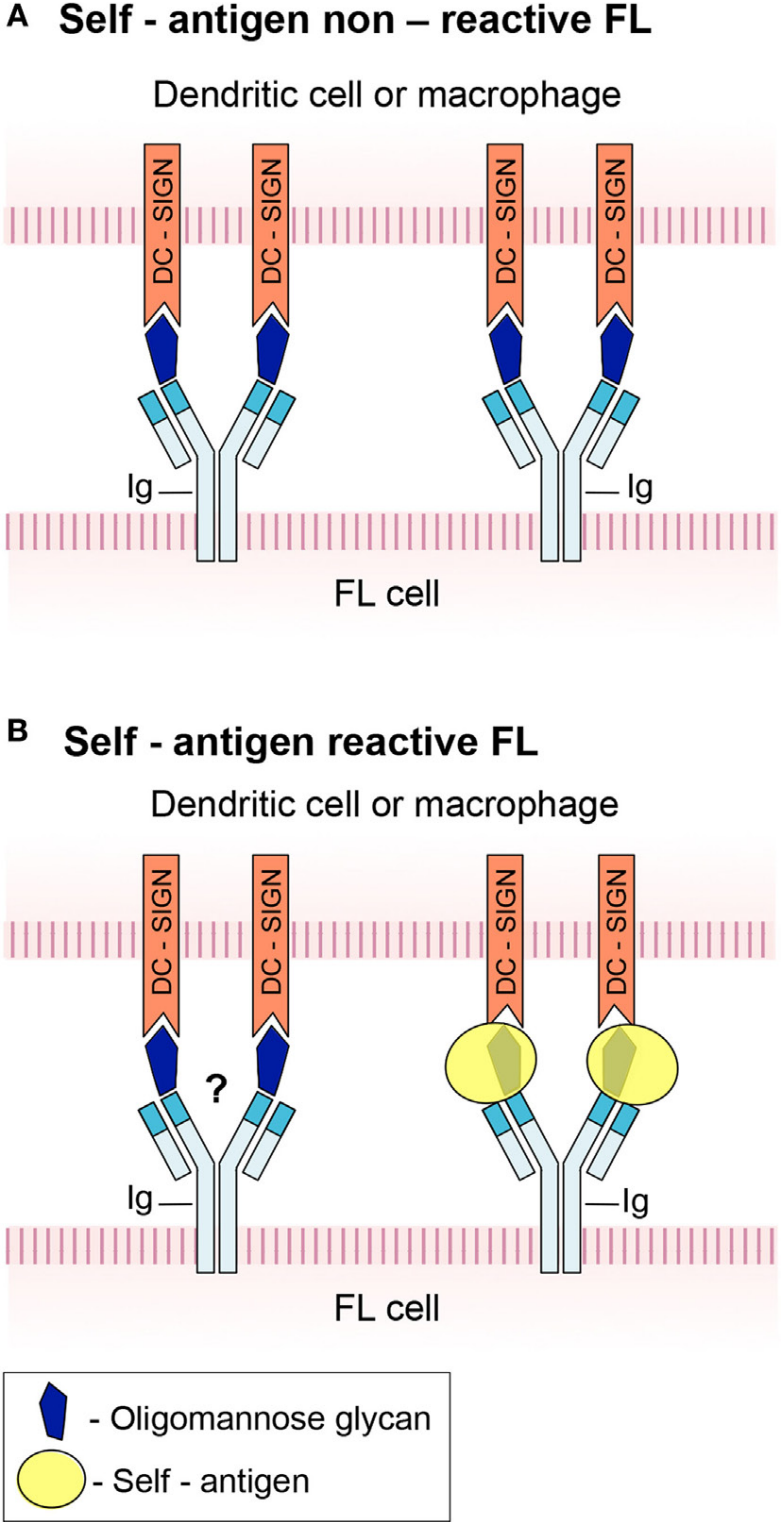
A schematic model of B-cell antigen receptor (BCR) engagement in follicular lymphoma (FL). **(A)** FL in which high-mannose glycan in the immunoglobulin (Ig) variable region prevents binding of cognate autoantigen to the BCR. The glycan interacts with DC-SIGN expressed by dendritic cells and macrophages, hence triggers FL cell activation. **(B)** FL in which the BCR is capable of antigen recognition and signaling despite the presence of a glycan in its active site. It is not clear whether concurrent interaction of the high-mannose glycan with DC-DIGN takes place.

There is a dispute regarding the Ig isotype that can bind lectin and trigger activation of FL. According to Linley et al. (14), activation by DC-SIGN occurred in both IgM^+^ and IgG^+^ FLs, while Amin et al. (15) reported that only IgM^+^ FL cells responded significantly to the lectin. The reason for this discrepancy is unclear. BCRs of IgG^+^ FLs are more commonly self-reactive compared to those derived from IgM^+^ FLs (11). It may, therefore, be speculated that although IgG^+^ FLs contain inserted N-glycosylation sites similarly to IgM^+^ FLs, lectin-mediated activation is mostly crucial for non-self-reactive FLs (mainly IgM^+^ tumors) that do not receive survival signals through autoantigen binding to the BCR. Nonetheless, lectin–BCR interactions may still play a role in pathogenesis of IgG^+^ FL despite their dispensability by augmenting autoantigen-specific activation. Such a model is illustrated in Figure 1. In any case, the findings that lectins within the microenvironment promote tumor survival through antigen-independent interactions with the BCR, at least in some FL subsets, are of clinical importance because they may provide new therapeutic targets (see below).

## Chronic Lymphocytic Leukemia (CLL)

Chronic lymphocytic leukemia, the most frequent leukemia in Western countries, is a malignancy of CD5^+^ mature-appearing B lymphocytes. Most tumors express IgM rather than class-switched isotypes. The malignancy is divided into two major subsets based on the mutational status of the Ig heavy-chain variable region genes. One is the unmutated CLL (U-CLL) that originates from a pre-germinal center B cell before initiation of somatic hypermutation. The second is the mutated CLL (M-CLL) that arises from a post-follicular B cell after accumulation of mutations due to somatic hypermutation. The two subsets behave differently, with U-CLL being more aggressive and generally having worse prognosis than M-CLL (24, 25). Similar to FL, expression of surface Ig is rarely lost in CLL, indicating its key role in tumor survival and growth. Evidence of restricted repertoire and biased use of certain Ig variable region genes, and findings that the BCR in CLL can bind some autoantigens and bacterial antigens, suggest a role for antigen engagement in selection of B-cell clones and leukemogenesis (26–29). Recognition of autoantigens followed by cell activation is mostly a feature of U-CLL cells, although few M-CLL Igs are reactive as well (29). *In vitro* ligation of the BCR by anti-μ heavy chain antibodies induced signaling that was significantly greater in U-CLL than in M-CLL (30). Evidence suggests that continuous engagement of surface Ig by autoantigens *in vivo* results in constitutive BCR signaling and proliferation of CLL cells, mainly of the U-CLL subset (31). In contrast, most M-CLL cells demonstrate anergy, which is manifested by weak signaling through the BCR and low cell proliferation (32). These findings provide at least one clue to the higher aggressiveness and worse prognosis of U-CLL.

Surface IgM of CLL exists in two forms that differ in their N-glycosylation patterns in the heavy chain constant region (33). One form carries mature complex glycans similar to normal B cells. The other carries immature high-mannose glycans that are normally restricted to the ER and absent from the cell surface. Both forms can mediate signaling in response to cross-linking with anti-μ heavy chain antibodies. Krysov et al. suggested that glycan modification is a consequence of antigen receptor engagement (33). They based their supposition on findings that U-CLL cells express higher proportion of mannosylated surface μ chains compared to M-CLL, that the high-mannose glycoform is reverted *in vitro* to the mature complex form, and that μ chains of normal B cells convert their glycan type from a complex to a high-mannose form in response to continuous ligation of surface IgM by anti-μ heavy chain antibodies. Thus, they proposed a model in which exposure to antigen results in downregulation of surface IgM by endocytosis in both U-CLL and M-CLL. The endocytic events lead to modulation of the μ-chain glycans, followed by loss of the mature complex form and retention of the immature high-mannose form, a process that is more evident in U-CLL due to continuous response to antigen (34). It should be noted that although extensive endocytosis may indeed lead to reduced surface expression, molecular mechanisms that alter glycosylation pathways following activation and endocytosis are not known. In addition, previous studies reported that the low expression of surface IgM in CLL results from intrinsic defects in glycosylation and folding of μ heavy chains and CD79a chains that lead to their retention in the ER (35). As a result of persistent cell activation, a fraction of these immature molecules may leave the ER and reach the plasma membrane either *via* a Golgi-dependent transport pathway, which is too fast or in which glycosylation sites are inaccessible to the appropriate enzymes, or *via* one of the established unconventional Golgi-independent transport pathways (4).

The higher expression level of the high-mannose glycoform in U-CLL compared to M-CLL and the similarity to its expression in FL suggest a functional significance for this glycan in CLL. In contrast to FL, mechanisms underlying its involvement in the pathogenesis of CLL have not yet been demonstrated. However, it is reasonable to assume that the high-mannose glycan interacts with environmental lectins, as shown for FL. It has been suggested that affinity of the BCR in the unmutated U-CLL subset is relatively low due to the lack of hypermutation and receptor affinity-maturation (34). It may, therefore, be speculated that lectin interactions with the mannosylated BCR of U-CLL augment activation and proliferation triggered by low-affinity BCR interactions with its specific autoantigen. Additionally, they may provide basal tonic signals that are indispensable for survival and known to be transduced by surface IgM in normal B cells. BCR–lectin interactions may also be involved in pathogenesis of M-CLL cells. The latter express very low levels of surface IgM and they show a highly reduced response to antigen due to anergy (32). However, even though antigen-induced activation is highly reduced or absent in M-CLL cells, they depend on tonic signals for survival. Interaction of the mannosylated BCR with environmental lectins may provide these tonic signals.

## Other B-Cell Malignancies

Acquisition of N-glycosylation sites in Ig variable region genes during somatic hypermutation is not unique to FL. It has also been detected in other germinal center-associated lymphomas, specifically in subsets of diffuse large B-cell lymphomas and Burkitt’s lymphomas (16, 21). Although these malignancies often show ongoing somatic mutation, there was no correlation between acquisition of novel glycosylation sites and levels of somatic mutation activity. Thus, some diffuse large cell lymphomas and Burkitt’s lymphomas with low mutational activity expressed the novel sites, while mucosa-associated lymphoid tissue lymphomas showing high mutational activity had low frequency of these sites. The lack of correlation suggests that tumor environmental factors are involved in positive selection of clones that are already mutated. Such factors could be stromal lectins that interact with acquired oligosaccharides in the mutated BCR. It should be noted that according to Zabalegui et al., N-glycosylation sites are inserted uniquely in FL, not in B-cell malignancies other than FL (19). This discrepancy may be accounted for by the small number of patients analyzed by Zabalegui et al.

Pre-B cells express surface pre-BCR, which is composed of μ heavy chains and surrogate light chains instead of the conventional Ig light chains. The pre-BCR governs antigen-independent proliferation and differentiation during B-cell development. Studies on distinct functions of the pre-BCR surrogate and heavy chains led to the following model: while signaling by the μ chains is sufficient to induce basal tonic signals that result in survival and differentiation of pre-B cells, the surrogate light chains enhance the signal and induce proliferation and clonal expansion of pre-B cells that produce functional μ chains (36–40). In contrast to the established critical involvement of the BCR in growth and survival of malignant B cells, a role for the pre-BCR in malignant pre-B cells remains unknown. Surprisingly, it has been demonstrated that all oligosaccharide moieties of μ chains in the pre-BCR of murine malignant pre-B cells are of the high-mannose type (41). The pre-BCR in these cells reaches the cell surface by a non-conventional transport pathway and is expressed on the surface in low levels (41, 42). This is reminiscent of the BCR μ heavy chains in CLL, suggesting possible involvement of the pre-BCR in pathogenesis.

It is worth mentioning that acute lymphoblastic leukemia (ALL) cells showed increased binding of the C-type lectins DC-SIGN and L-SIGN, indicating altered glycosylation patterns in this malignancy (43). High DC-SIGN and L-SIGN binding to B-cell ALL correlated with poor prognosis, suggesting involvement of this interaction in pathogenesis. While CD15 (Lewis X) was shown to be the ligand for DC-SIGN in some B-ALL patients, this is not the case in most patients (43). Hence, other ligands play a role in the interaction of ALL cells with DC-SIGN. Candidate ligands could potentially include altered high-mannose glycans of Ig heavy chains, as in the malignancies described above, or other surface glycoproteins. The hypothesis, that altered glycans of non-Ig surface components may be involved, is based on the demonstration that exceptional glycoproteins such as CD45 in immature thymocytes may uniquely display high-mannose glycans (44, 45).

## Therapeutic Targeting of Altered Glycans

Lectin binding to the BCR of FL triggers survival signaling pathways similar to those demonstrated for antigen-engaged normal BCR. Hence, interference with BCR signaling may be an effective therapeutic approach for lectin-driven malignancies, as already suggested for antigen-driven B-cell tumors. Indeed, both DC-SIGN-mediated BCR signaling and cross talk of M2 macrophages with FL cells were inhibited *in vitro* by BTK and SYK inhibitors (14, 15), revealing their therapeutic potential. Ongoing clinical trials are currently analyzing the therapeutic efficacy of signal transduction inhibitors in FL patients and in other B-cell malignancies that depend on BCR signaling (46–48).

As interaction of lectins with mannosylated surface Ig is critical for survival and growth of FL and some other lymphomas, targeting this interaction may provide an additional therapeutic strategy. Blockade of lectin–BCR interactions may be inspired by reagents that are currently being developed to combat human immunodeficiency virus, since envelope glycans of this virus are almost entirely of the oligomannose type (49). These reagents include antibodies against high-mannose glycans (50, 51) and mannose-based oligosaccharide mimics or non-carbohydrate glycomimetics that act as competitive inhibitors of lectin–glycoprotein interactions (52). Interestingly, Schneider et al. demonstrated that lectins from opportunistic bacteria, which present a threat for immunocompromised patients, bind to the acquired high-mannose glycans of FL, and stimulate the malignant cells (12). Treatment with anti-mannose antibodies or with glycomimetics may, therefore, disrupt not only tumor interaction with environmental lectins but also its interaction with opportunistic pathogens. Furthermore, treatment with antibiotics to combat the opportunistic bacteria may additionally attenuate tumor growth due to disruption of bacterial interactions with the tumor.

## Author Contributions

NH designed and wrote the review. JH revised the manuscript and the figure.

## Conflict of Interest Statement

The authors declare that the research was conducted in the absence of any commercial or financial relationships that could be construed as a potential conflict of interest. The reviewer AJP and handling editor declared their shared affiliation and the handling editor states that the process nevertheless met the standards of a fair and objective review.
